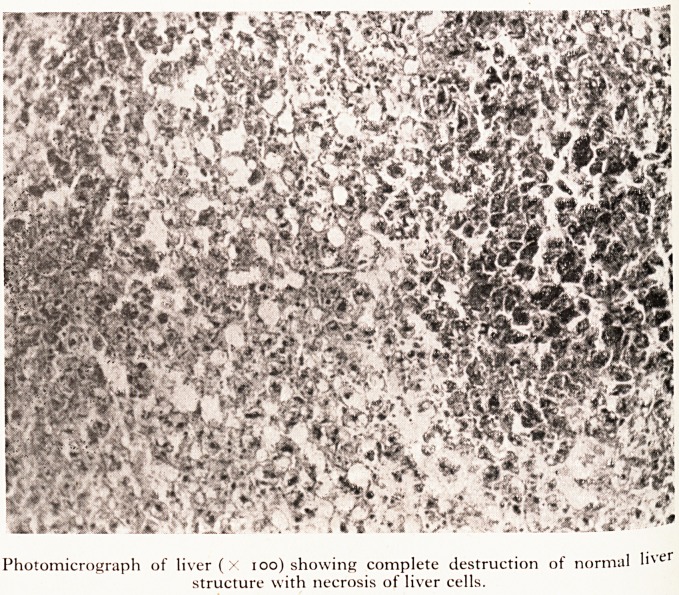# Acute Necrosis of the Liver in Pregnancy

**Published:** 1957-10

**Authors:** T. F. Hewer


### ACUTE NECROSIS OF THE LIVER IN PREGNANCY

*A Clinical Pathological Conference held at Canynge Hall on 23rd October, 1956*

CHAIRMAN: PROFESSOR T. F. HEWER

*Dr. G. F. Jolly:* This patient, a woman, aged 27, who was normally domiciled in Northern Ireland, first came to Bristol in February, 1956, when she was five weeks pregnant; she had had no previous children. She was seen at Southmead Hospital at the end of that month by Dr. P. Phillips. The date of her last menstrual period was given as 7th January, 1956 from which it was calculated that she would be due on 14th October. The height of the fundus corresponded with these dates. Her blood pressure and urine were normal, there was no oedema, and her weight was 7 stone. A few days later there was slight leucorrhoea for which acetarsol pessaries were prescribed. Regular ante-natal supervision was carried out by her general practitioner. Her blood pressure remained at 120/70 mm. Hg. or less and there was no albuminuria.

On 8th August, at the 30th week of gestation her blood pressure was found to have risen to 140/90 mm. Hg. and she had swelling of the feet and hands. She was referred again to Dr. Phillips who advised more rest and suggested Sodium amytal gr. 3 nightly. After this there was no further rise in blood pressure, no albuminuria and only occasional swelling of the feet and ankles.

On 17th September, she was admitted to Southmead Hospital, 36 weeks pregnant and thought to be in early labour. There had been backache for ten days and some pain before micturition. In appearance she was a thin sallow girl with greying hair, carious teeth and a marked squint. The heart, lungs and urine were normal, the blood pressure was 160/100 mm. Hg. and the fundus height corresponded to 34 weeks gestation. The presentation was a vertex, L.O.A., the head was free and the foetal heart was heard. She was confined to bed and given Sodium amytal gr. 3 six-hourly. During the next few days her blood pressure varied between 120/70 and 160/100 mm. Hg. and daily urine testing revealed no sugar or albumin, until 24th September, when a trace of albumin was found.

On 25th September she vomited twice and complained of sharp pains in the right flank, worse on lying down. There was no abdominal tenderness. The next day she fell out of bed but made no complaints. On 27th September when seen by Dr. Phillips on the morning round, she was very drowsy with sluggish reactions. She was, however, quite rousable and no abnormal physical signs were found. All sedation was stopped. The next day she was still very drowsy but taking fluids well. Her blood pressure was unchanged and the urine was normal. On 29th September she was slightly less drowsy but looked ill and was now definitely jaundiced Hb. 87%, W.B.C.'s 10,000 per cu. mm., serum bilirubin 5·2 mgms. per 100 ml. She was seen by Dr. H. G. Mather who agreed with Dr. Phillips that delivery should be expedited on account of impending liver failure. Accordingly that same evening a surgical induction was carried out. I found the cervix to be one finger dilated. The fore-waters were ruptured and the head, which was presenting, was pushed into the pelvis. A small septum in the lower third of the vagina was broken during the process. Labour soon became well established but the foetal heart which had been heard when the membranes were ruptured could be heard no longer. She laboured steadily during the night, her only sedation being 100 mgms. of Pethidine given by way of the intravenous drip which had been set up. At 8.45 a.m. the following morning the cervix was fully dilated but the head was high and lying transversely. There was no advance after one and a half hours and so Kielland's forceps were used for rotation and extraction under a low spinal anaesthetic, a caudal block having been unsuccessful. It was a difficult delivery as not only could the presenting part not be seen at the vulva but the pelvis was android in shape with prominent spines and a narrow supra-pubic angle. A fresh, stillborn, male infant weighing 6 lbs. was delivered. There was no excessive bleeding.

After delivery the systolic blood pressure fell to 60 mm.Hg., the diastolic pressure being unrecordable. By mid-afternoon with recovery from the spinal anaesthetic and the administration of pressor agents the blood pressure rose to 120/70 mm. Hg. but her condition steadily deteriorated despite continuous intravenous therapy and blood transfusion and the use of nor-adrenaline and 32 hours after delivery she died.

On the last day of life the blood urea was 64 mgms. %, serum alkaline phosphatase 84 units, serum bilirubin 14·7 mgms. per 100 ml., thymol turbidity 2 units and serum proteins 5·3G per 100 ml. (albumin 3·0, globulin 2·3).

*Question:* Had any drugs been taken which might have had a toxic effect on the liver?

*Dr. Jolly:* Apart from the Sodium amytal the only drug which there is any record of her having received before the onset of jaundice is acetarsol, which was administered early in pregnancy in the form of pessaries.

*Dr. H. G. Mather:* Some blood tests were taken at another clinic earlier in pregnancy. I mention this because there is a possibility that this might be a case of so-called "syringe hepatitis".

*Dr. G. E. F. Sutton:* Was the urine examined for the presence of leucine and tyrosine crystals? These are sometimes found in cases of acute necrosis of the liver.

*Dr. F. J. W. Lewis:* These were not observed but even in severe cases of liver necrosis they are not always found.

*Dr. N. J. Brown (presenting the post-mortem findings):* This was the body of a well-nourished young woman. There was moderately deep jaundice and a few purpuric spots were present on the anterior abdominal wall and on the legs. There was no oedema. There were lacerations of the cervix and vagina. The uterus was partially involuted; it had a shaggy haemorrhagic lining to which a piece of membrane was still adherent. The ovaries and Fallopian tubes were normal.

Not only was the skin jaundiced but so were all the organs. The endothelium of the aorta and the heart valves were stained bright yellow and there was a slight excess of dark yellow fluid in all the serous cavities. The cause of the jaundice was found, not unexpectedly, in the liver which presented a really remarkable sight. It is illustrated in Plate XII which shows three livers and one spleen. The uppermost of the three livers is that of a normal adult, the lowermost that of this woman's stillborn baby. The centre liver is that of the patient and it will be seen at once that its size is nearer that of the baby than the adult. Its weight was in fact only about one third of the normal. It was yellowish red in colour and extremely soft and flabby. The reason for this is that it was completely and absolutely *dead.* So, of course, was the patient, but the liver had died several days before she died. Histologically (Plate XIII) it contained not one normal liver cell. The picture is that of complete necrosis of the liver cells with only the fibrous framework and bile ducts surviving. There is no cellular inflammatory reaction. The only thing that one can say is that the cells in the central zones appear, if anything, rather more dead than those at the periphery of the lobules. The appearances are those of an acute necrosis of the liver, or, to use the older term, of acute yellow atrophy.

The spleen was firm and dark red. The lungs showed congestion and oedema. The adrenal glands and thyroid showed the hyperplasia normally found in pregnancy. The thymus was easily recognisable. Lastly, two rather unexpected features were found in the kidneys and the pituitary gland.

The kidneys showed the slight physiological hydronephrosis of pregnancy. They were yellow in colour and slightly swollen with blurring of the cortico-medullary pattern and congestion of the boundary zone. Histologically there was necrosis of the second convoluted tubules with the formation of granular casts. The glomeruli appeared ischaemic. There was therefore an acute renal tubular necrosis.

The pituitary showed no obvious gross abnormality but histologically this also showed patchy necrosis of the anterior lobe.

### PLATE XII

(*Top*) Normal adult liver. (*Centre*) Patient's liver.
(*Bottom*) Baby's liver. (*Right*) Patient's spleen.

### PLATE XIII

Photomicrograph of liver (× 100) showing complete destruction of normal liver structure with necrosis of liver cells.

To sum up, therefore, there was:

(*a*) Acute necrosis of the liver.
(*b*) Acute necrosis of the renal tubules.
(*c*) Acute necrosis of the pituitary.

Now let us try to relate these findings to the clinical history. I think the last two conditions—the renal tubular and pituitary necrosis—were secondary and almost terminal events which were brought about by the prolonged fall in blood pressure following delivery. They are well known complications of such hypotensive episodes during and after childbirth and they do not further concern us here. What we are principally concerned with in this case is the acute necrosis of the liver which gave rise to the severe and fatal hepatic failure. This of course, is also known to be sometimes associated with pregnancy but is very rare. In the seven years that I have been at Southmead I have seen only one other fatal case.

The cause of the condition is a matter for discussion. The possibilities are:

(*a*) Fulminating infective hepatitis.

(*b*) Homologous serum hepatitis.

(*c*) Nutritional deficiency.

(*d*) Drug reaction.

The clinical course of the present case is unlike that of infective hepatitis which not infrequently occurs in pregnancy and is usually a relatively mild disease. There is a possibility that it might be homologous serum jaundice as although she had had no blood transfusions there was a history of a diagnostic intravenous puncture some months previously and it is possible that the virus might have been introduced then. In favour of a virus aetiology of the present condition is the fact that the necrosis is more severe in the centrilobular zones which is the usual site of maximum damage in virus hepatitis. Against it however, is the complete absence of any inflammatory cellular reaction in the liver.

It has been suggested that acute hepatic necrosis of pregnancy might be due to nutritional deficiency. Himsworth and Glynn have shown that if rats are fed on a diet deficient in the amino-acid cystine they develop sudden acute necrosis of the liver. The very similar sudden onset of the disease in this case makes one wonder whether a similar mechanism might not be involved. There was no eivdence of any general state of malnutrition but it is not impossible that the demands of a growing foetus on a pregnant woman coupled perhaps with a somewhat capricious appetite might lead to a specific protein or amino-acid deficiency although I admit that in this case this is pure speculation.

Lastly, could this be a toxic effect of some drug? The only difficulty here is that the only drug administered in the weeks preceding the jaundice was sodium amytal which, as far as I know, is not supposed to have any toxic effects on the liver. The only other drug she had was acetarsol in the form of pessaries early in pregnancy.

There is, of course, the possibility that the liver necrosis might have been due to a combination of more than one of these factors. An attack of virus hepatitis might for instance have been made much worse by amino-acid deficiency, or a superimposed drug reaction, and all the time we have to remember that this was a pregnant woman at term when metabolic processes and body reactions as a whole are not quite the same as in the ordinary adult.

*Prof. T. F. Hewer:* Was there any fat in the liver?

*Dr. Brown:* I have not done a frozen section but in the paraffin sections it looks as if there is some.

*Dr. Sutton:* In the old days we used to see many more of these cases and it appears that it is much less common nowadays than it used to be. Acute yellow atrophy is a good name for it. It always occurred suddenly "out of the blue" and the patient was dead in a few days with the liver shrivelled up to a fraction of its normal size. I think dietary deficiency must have something to do with it and of course nutritional deficiency used to be much more common than it is now which would explain why we don't see it so often these days.

*Question:* If the disease is due to protein deficiency would it not be logical to treat it by a high protein diet?

*Dr. H. G. Mather:* No. It is possible that a high protein diet might have some effect in *preventing* liver necrosis but it would be quite wrong to treat it with high protein as the liver is so damaged that protein metabolism is grossly interfered with and a great deal of the harmful effects of liver failure is due to the accumulation of amino-acids in the blood.

*Dr. T. M. Abbas:* This case presents many interesting problems. In the first place I do not agree that a woman with a blood pressure of 190/105 is, as is stated in the summary sheet, in a state of "mild toxaemia".

I should like to ask one question—We have been told that this patient had acetarsol therapy for her vaginal discharge, can we be assured that this was not taken orally?

*Dr. Jolly:* I can give no such assurance. I can only say that it was prescribed in the form of pessaries.

*Dr. Mather:* I think that the toxic effects which were previously ascribed to arsenic when given by injection are now generally accepted as being, in fact, homologous serum hepatitis caused by a virus due to the use of imperfectly sterilised syringes. I do not think there is much evidence that arsenic given by mouth has any toxic effect on the liver.

*Prof. Hewer:* It is difficult to say in this case whether there was a massive necrosis of the liver from the start or whether it is a zonal necrosis which has become confluent.

*Dr. Brown:* There was, as I showed just now, more complete necrosis in the centres of the lobules which suggests that there was a zonal necrosis to start with. This would fit in with homologous serum hepatitis.

*Dr. J. E. Cates:* Was the baby's liver examined?

*Dr. Brown:* Yes, and it appeared entirely normal. One might argue that this is against a virus infection but even if a mother has hepatitis late in pregnancy the baby is very rarely affected.

*Prof. Hewer:* In any disease of pregnancy the baby always gets the best of it.

*Dr. Cates:* What was this woman fed on during the fortnight preceding delivery?

*Dr. Jolly:* She had her normal diet despite her toxaemia. There was no reduction in diet.

*Dr. Brown:* Did she finally reduce her intake on account of vomiting?

*Dr. Jolly:* I believe she was feeding well. She did not start vomiting until 25th September—four days before the jaundice appeared.

*Dr. Sutton:* This case does not sound like one of virus hepatitis. When we used to see a lot of these cases we found that it was necessary to evacuate the uterus as soon as possible; otherwise they do not survive.

*Prof. Hewer:* The prognosis depends entirely on how much liver is left alive. The capacity of the liver cells to regenerate is tremendous but if too much is destroyed the patient must die.

## Figures and Tables

**Figure f1:**
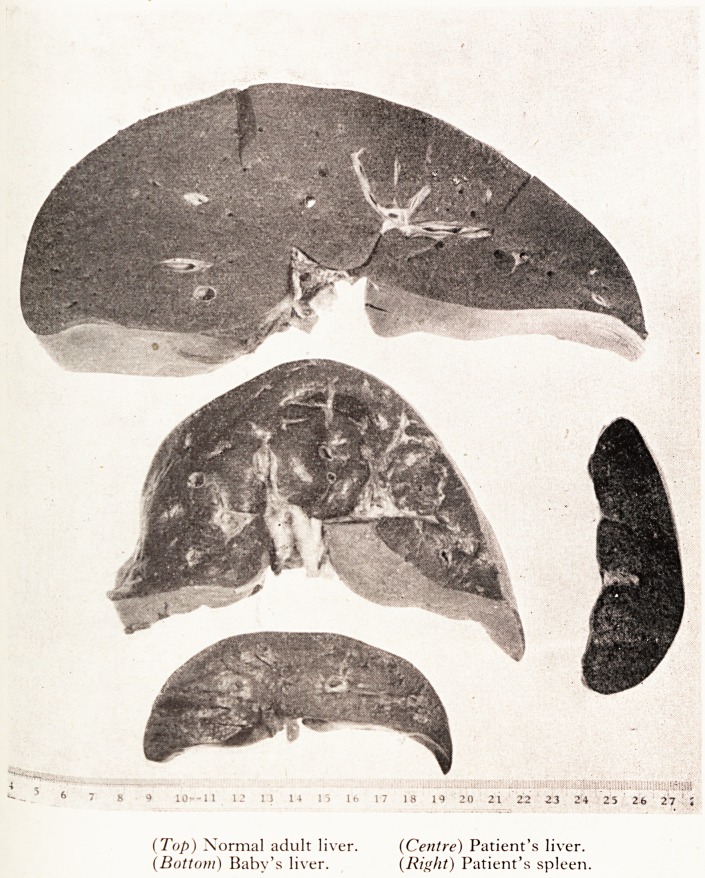


**Figure f2:**